# Feasibility of long-range telesurgical robotic radical gastrectomy in a live porcine model

**DOI:** 10.1097/JS9.0000000000002151

**Published:** 2024-11-22

**Authors:** Daryl Kai Ann Chia, Koichi Suda, Wei-En Ho, Bu Sung Lee, Kazumitsu Suzuki, Susumu Shibasaki, Tatsuhiko Harada, Jun Murai, Masafumi Oe, Hirochika Asai, Takashi Tomine, Hirotaka Sato, Masahiro Yoro, Masanao Ohashi, Hiroaki Kitatsuji, Simon Peter Green, Ying Xiong, Asim Shabbir, Davide Lomanto, Jimmy Bok Yan So, Guowei Kim, Ichiro Uyama

**Affiliations:** a Department of Surgery, National University Hospital, Singapore, Singapore; b Centre for Obesity Management and Surgery, National University Hospital, Singapore, Singapore; c Department of Surgery, Fujita Health University, Aichi, Japan; d Collaborative Laboratory for Research and Development in Advanced Surgical Intelligence, Fujita Health University, Aichi, Japan; e Ministry of Health Holdings, Singapore, Singapore; f Singapore Advanced Research and Education Network (SingAREN), Singapore, Singapore; g School of Computer Science and Engineering, Nanyang Technological University, Singapore, Singapore; h Department of Advanced Robotic and Endoscopic Surgery, Fujita Health University, Aichi, Japan; i WIDE Project, Japan; j National Astronomical Observatory of Japan, Mitaka, Tokyo, Japan; k Asia-Pacific Advanced Network - Japan (APAN-JP), Japan; l Medicaroid Asia Pacific Pte. Ltd., 9 Tampines Grande, Asia Green, Singapore, Singapore; m Department of Product Marketing, Medicaroid Corporation, Hyogo, Japan; n Medicaroid Corporation, Hyogo, Japan; o Department of Surgery, National University of Singapore, Singapore, Singapore; p Collaborative Laboratory for Research and Development in Advanced Surgical Technology, Fujita Health University, Aichi, Japan

**Keywords:** anastomosis, animals, computer communication networks, gastrectomy, hinotori Surgical Robot System, models, preclinical studies, remote operations, robotic surgical procedures, surgical

## Abstract

**Background:**

Telesurgery has been made increasingly possible with the advancements in robotic surgical platforms and network connectivity. However, long-distance transnational complex robotic surgeries such as gastrectomy have yet to be attempted.

**Methods:**

Multiple transnational network connections by Science Innovation Network (SINET), Japan Gigabit Network (JGN), and Arterial Research and Education Network in Asia-Pacific (ARENA-PAC) were established and tested by multiple surgeons in a dry box model. Surgeons’ perceptions of the different networks were recorded. Three robotic radical D2 gastrectomies in live porcine models were performed at a hospital in Toyoake, Japan, by a surgical team in a hospital in Singapore ~5000 km away, using the hinotori Surgical Robot System (Medicaroid Corporation).

**Results:**

The live porcine robotic gastrectomies were all completed in under 205 min with no intraoperative complications. From the different networks that were tested, the differences in latency ranged from 107 to 132 ms and did not translate to any significant differences in surgeon timings and perceptions.

**Conclusions:**

Transnational telesurgical radical D2 gastrectomy is feasible in a porcine model. There is no appreciable difference between surgeon performance and perception with network latencies of 107–132 ms. Long-range telesurgery as clinical practice may become possible in the future.

## Introduction

Highlights

- Three telesurgical robotic gastrectomies were performed successfully on live porcine models.
- Between Fujita Health University, Japan and National University Hospital, Singapore.
- Network support with high-speed and low-latency networks.
- Dry-test procedures to evaluate various network latencies performed by surgeons.
- Network latencies of 107–132 ms had no impact on surgeon performance and perception.

Telesurgery has been classified as telementoring (providing instructions remotely), telesurgical support (use of a robot to assist with surgery), and full telesurgery (entire surgery performed from a remote location)^1,2^. In recent years, the advancements in robotic surgery platforms and network infrastructure, alongside rapid globalization, have made telesurgery increasingly appealing and applicable. To date, robotic telesurgery has been reported to be feasible for robotic cholecystectomy, fundoplication, colectomy, and hernia repairs^3^ but has largely remained within the same country/continent with the exception of Marescaux’s transcontinental robot-assisted laparoscopic cholecystectomy in 2001^4,5^.

To accomplish safe transnational telesurgery, surgical information must be transmitted rapidly over long distances securely and via a stable network with minimal latency. The risk of misjudgment, overshooting, and failure of the operator to compensate for these in the event of an unstable and high-latency network are major concerns that could increase operative risk. This is particularly pertinent with complex procedures like a radical gastrectomy where the anatomy encountered is technically challenging and in close proximity to multiple major vascular structures^6^. Telesurgical distal gastrectomies have been reported in cadaveric and live porcine models over relatively short distances but have not been attempted as a long-range transnational remote telesurgical procedure^7,8^.

Thus, this study aimed to evaluate the feasibility of performing remote robotic gastrectomy in a live porcine model via a long-range transnational network between two hospitals in Japan and Singapore over a distance of ~5000 km using the hinotori Surgical Robot System (Medicaroid Corporation).

## Methods

### Robotic platform

**Figure 1:**
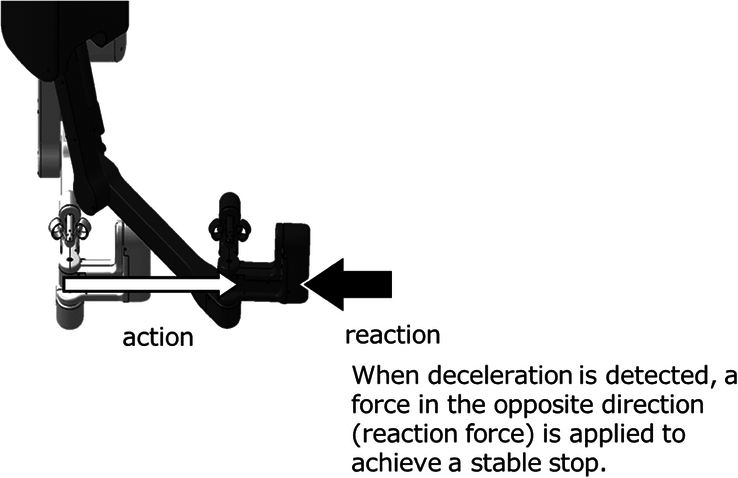
Software update on hinotori Surgical Robot System aimed at monitoring the operation speed of the hands control.

The study was performed on the hinotori Surgical Robot System developed by Medicaroid Corporation, Kobe, Japan, and has previously been successfully utilized in various urological, gastroenterological and gynecological procedures^9–12^. It comprises a Surgeon Cockpit, vision unit and operation unit. The operation unit has four arms with eight axes, providing more flexibility in joint movement compared to the conventional da Vinci’s robotic system^12^. The surgeon’s movements are transmitted from the Surgeon Cockpit to the operation unit. In August 2023, major software and hardware upgrades for the hinotori Surgical Robot System allowed for monitoring the operation speed of the hands control; when a deceleration is detected, a force (reaction force) is applied in the opposite direction of the operation, thereby reducing the feeling of instability (e.g. floating sensation or oscillation) during the operation (Fig. 1). In this study, the Surgeon Cockpit was set up in Singapore, ~5000 km apart in a straight line from the operation unit located in Toyoake, Japan.

### Network connection

**Figure 2:**
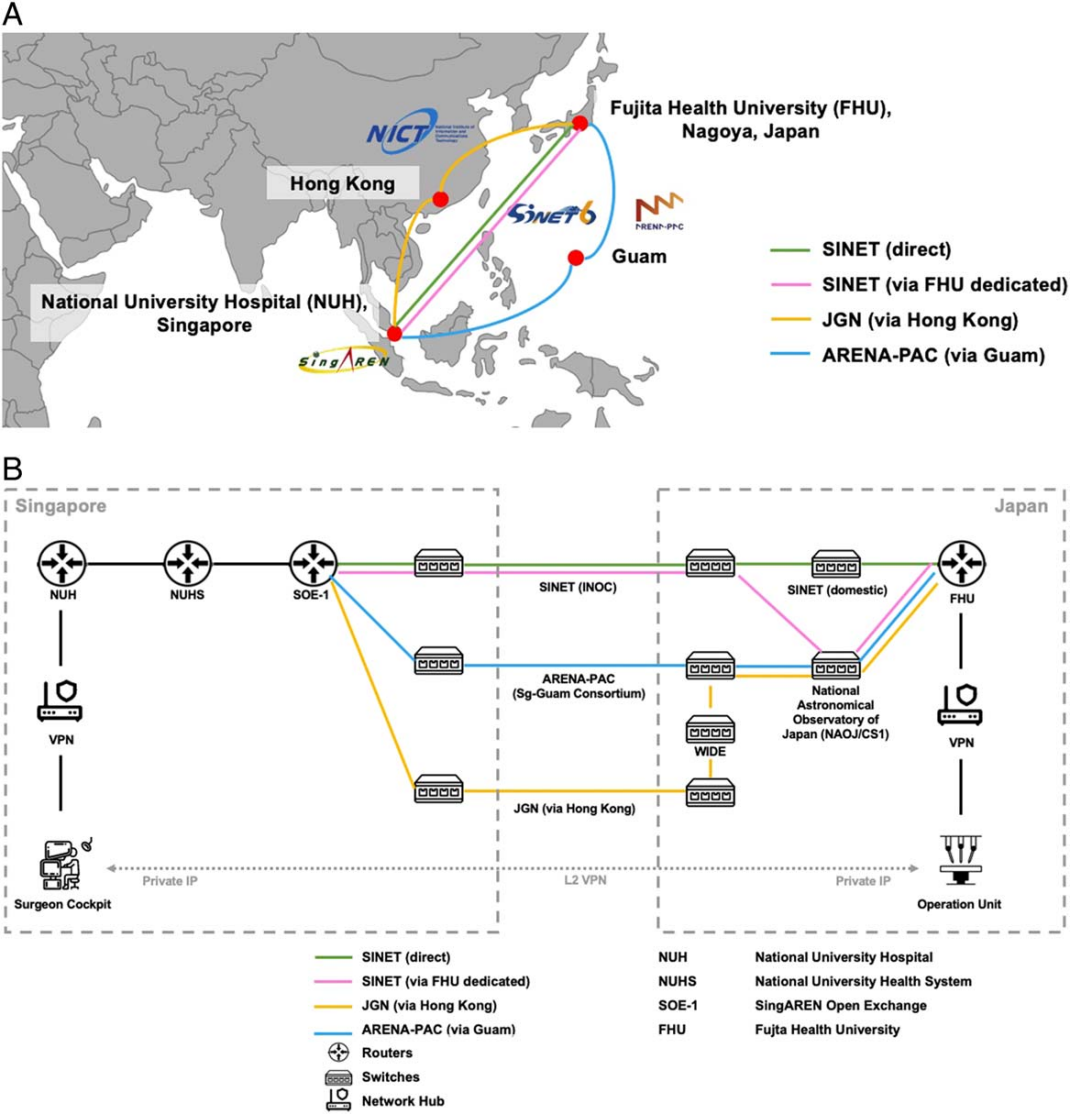
(A) Global Network Set-up. Four geographically distinct network links were set up between Research and Education Networks. Two network links ran through SINET, operated by Japan’s National Institute of Informatics (NII). The JGN network link, by Japan’s National Institute of Information and Communication Technology (NICT), transmitted data via Hong Kong. ARENA-PAC, operated by the WIDE project, transmitted data via Guam. (B) The two robot pods (Surgeon Cockpit and operation unit) are connected via the campus networks. Within the pods, there are multiple devices and sensors with their IP address. The two pods communicate with each other via a series of routers and switches through a private IP protocol.

The network connection between the two robot pods, comprising the operation unit in Toyoake, Japan, and the Surgeon Cockpit in Singapore, was supported by SingAREN (Singapore Advanced Research and Education Network)^13^ and WIDE (Widely Integrated Distributed Environment) Project^14^. The two robot pods were linked via campus networks, which were then connected internationally via the Research and Education Networks (REN) that have been deployed under the Asia Pacific Ocean Network (APOnet) collaboration^15^. Multiple levels of redundancies and fail-safes were incorporated to ensure a resilient connection between both pods. Four networks – Science Information Network (SINET) (direct), SINET (via FHU dedicated), Japan Gigabit Network (JGN), and Arterial Research and Education Network in Asia-Pacific (ARENA-PAC) – were created across geographically diverse paths (Fig. 2A). The SINET (via FHU dedicated) network connection was composed of SINET [internet network operations center (INOC)] and FHU dedicated line [via National Astronomical Observatory of Japan (NAOJ)]. These connections between both robotic pods involved an extensive series of switches, routers, and hubs (Fig. 2B). Virtual private networks (VPNs) were set up such that the two pods were connected directly to ensure privacy and security. Real-time multimedia protocols were employed to minimize delays.

The total latency time between the Surgeon Cockpit in Singapore and the operation unit in Japan was defined as the sum of network and system delays. The threshold of total latency time was projected to be within 100 ms to ensure smooth task performance. Network delay was defined as the time taken solely for information transfer across the network. System delay included the processing time – encoding, decoding, and buffering of data by the telesurgical platform.

### Experimental setup

#### Standardized surgical tasks

The aim of the standardized trials was to evaluate individual surgeons’ objective performance and subjective perception of the various networks with differing latencies. Three attending upper gastrointestinal surgeons from Singapore participated in the standardized trials. The three attendings, with prior experience in performing robotic surgeries on the da Vinci robotic platform, had introduction and practice sessions on the hinotori for >2 h prior to the experiments.

**Figure 3:**
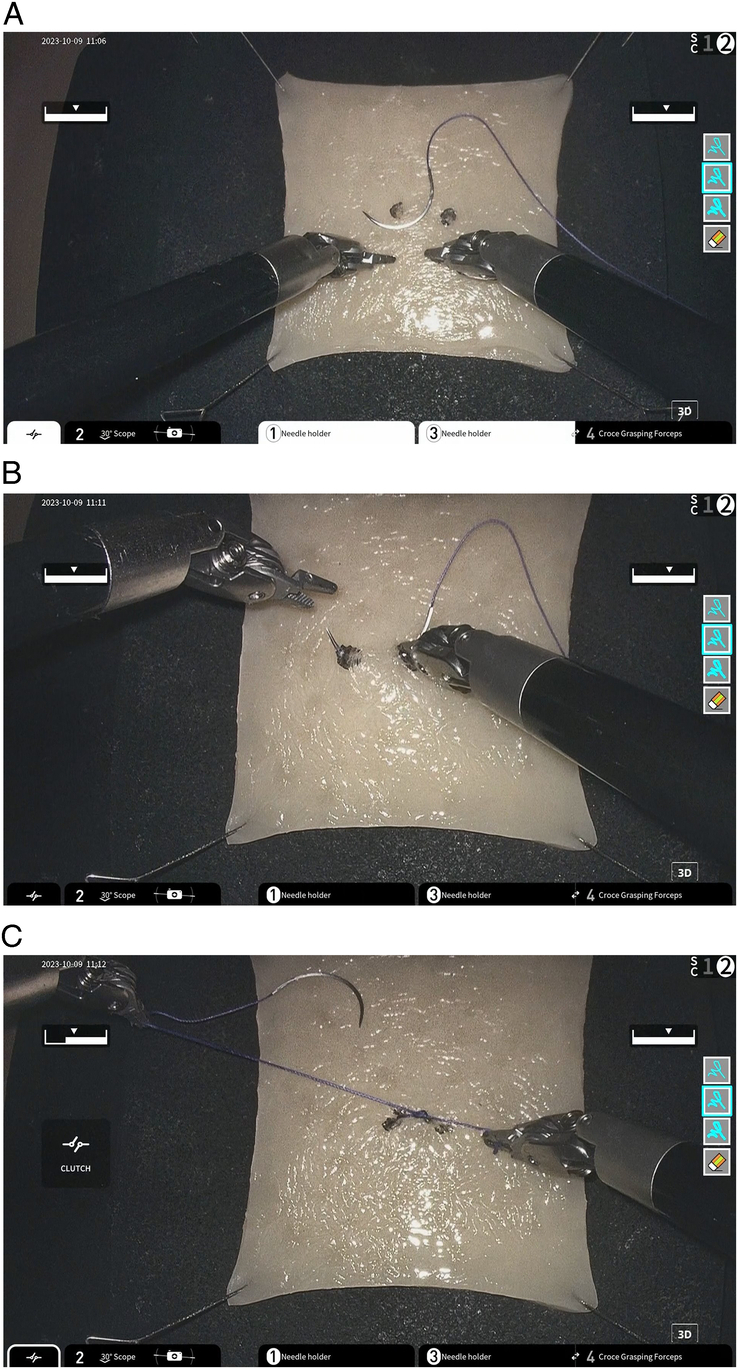
(A) Task set-up was with Ethicon VICRYL 3-0 SH-1. (B) Suture had to be passed through both black dots (C) Suture was tied down. Achieving the above was deemed as successful task completion.

The standardized tasks involved suturing and knot-tying on a synthetic suture training model, via the Surgeon Cockpit in Singapore to the operation unit in Toyoake, Japan. This involved driving the needle through two black dots and firmly securing the knot after (Fig. 3). The three surgeons performed the tasks on each of the international networks thrice. The surgeons were blinded to the order and the specific network that the procedure was running on (SINET (direct), SINET (via FHU dedicated), JGN, and finally ARENA-PAC). The time taken for task completion was measured for each attempt. After completing the tasks thrice on each network, the surgeons’ feedback on each network was evaluated with a questionnaire (Appendix Fig. 1, Supplemental Digital Content 1, http://links.lww.com/JS9/D572) focusing on ‘Delay’, ‘Stress’, and ‘Smoothness of Operation’. Rather than binary answers, responses were graded on a Likert scale of 0–10 to better evaluate a gradient of differing perception with better resolution. The goal was to elicit the level of discomfort experienced as well as evaluate the mental effort required at networks of various latencies, similar to other survey studies conducted^8,16^.

#### Fasotec model gastrectomy

Prior to the live porcine gastrectomies, each surgeon practiced a telesurgical gastrectomy on a synthetic training model for gastrectomy (Fasotec Co., Ltd)^17^. The dry gastrectomy model and operation unit was located in Japan, while the surgeon operated via the Surgeon Cockpit in Singapore.

#### Telesurgical gastrectomy in porcine models

A total of three telesurgical radical gastrectomy procedures were performed under general anesthesia remotely on live animals by upper gastrointestinal surgeons from both Japan and Singapore. The surgeons performed a standardized robotic distal gastrectomy with D2 lymphadenectomy and intracorporeal Billroth-I anastomosis from the hinotori Surgeon Cockpit in Singapore, while the operation unit and porcine model was stationed in Toyoake, Japan. This was done via the SINET (via FHU dedicated) network. The other three networks served as fail-safe networks in the event of a disruption on the SINET (via FHU dedicated) line.

The details of the surgical set-up for our telesurgical gastrectomy were similar to the previous experiment reported by Nakauchi *et al*.^8^ All the procedures were performed under the approval of the Institutional Animal Care and Use Committee at the hospital in Toyoake, Japan (APU22141-MD2)^18^. The handling of the porcine models was in accordance with the policies detailed by the Regulations for the Management of Laboratory Animals at the hospital in Toyoake, Japan. Three female 10–13 week-old White-Landrace (WL) specific pathogen-free porcine (ZPP, ZEN-NOH) were purchased and kept in a controlled room set at 20±5°C with 12 h light/dark cycles. The animals were allowed to eat and drink anytime, and were fasted 12 h prior to anesthesia to reduce vomiting. The animals were premedicated with intramuscular midazolam, medetomidine, and butorphanol, and subsequently induced with isoflurane by mask before intubation. Set-up and positioning were done with five trocars (including one port for an assistant surgeon) placed in the upper abdomen. The surgeon’s right hand controlled either a Maryland Bipolar Forceps or monopolar curved scissors connected to an AUTOCONTM II 400 Electrosurgical Unit, as well as a Universal grasp (Medicaroid Corporation). The surgeon’s left hand was in control of a Croce grasping forcep or fenestrated bipolar forcep (Medicaroid Corporation). The surgical procedure entailed gastrectomy with D2 lymphadenectomy and the creation of a Billroth-I anastomosis with linear staplers. Each animal was under the care of a veterinarian at all times and euthanized immediately after the completion of each procedure. The work has been reported in accordance with the Animals in Research: Reporting in Vivo Experiments (ARRIVE) guidelines (Supplemental Digital Content 2, http://links.lww.com/JS9/D573)^19^.

#### Statistical analysis

Statistical analysis was performed by using SPSS Statistics Version 28.0 (IBM SPSS Inc.). All data is presented as median (interquartile range). Kruskal–Wallis tests were used to compare the time to task completion and surgeon perceptions between the various networks, with the threshold for statistical significance set at *P*<0.05.

## Results

### Latency times

The total latency times by each network are presented in Table 1. SINET (direct) and SINET (via FHU dedicated) connections had the least delay, with both networks having a network delay of 77 ms. This was followed by JGN, with a delay of 94 ms, and finally ARENA-PAC, with a delay of 102 ms. The system delay was constantly 30 ms regardless of each network.

**Table 1:** Latency time for each network.

	SINET (direct)	SINET (via FHU dedicated)	JGN	ARENA-PAC
Network delay (ms)	77	77	94	102
System delay (ms)	30	30	30	30
Latency time (ms)	107	107	124	132

### Standardized surgical tasks

Images of the standardized suture ligation tests are seen in Figure 3. Tasks were timed for each attempt, and task failure was defined as failure to pass the needle through both black dots.

The median time taken for completion of the standardized tasks for each surgeon is presented in Table 2. On the SINET (direct) and SINET (via FHU dedicated), median times were 74.0 s (70.0–80.5 s) and 87.0 s (80.5–103.5 s), respectively. The median time for the JGN network was 87.0 s (70.5–122.0 s), and for the ARENA-PAC network was 74.0 (63.0–86.0 s). There was no difference in the median time taken to complete the tasks between each network (Table 2). Surgeon perceptions on the delay, stress, and smoothness of the standardized tasks on various networks were analyzed and represented in Table 3. There were no significant differences in surgeon perception scores across all networks in the domains of perceiving ‘Delay’, ‘Stress’, and ‘Smoothness’.

**Table 2:** Time taken for completion of standardized tasks.

Network	*N*	25th percentile	50th percentile (median)	75th percentile	Interquartile range	Kruskal–Wallis test (*P* value)
SINET (direct)	9	70.0	74.0	80.5	10.5	0.133
SINET (via FHU dedicated)	9	80.5	87.0	103.5	23.0	
JGN	9	70.5	87.0	122.0	51.5	
ARENA-PAC	9	63.0	74.0	86.0	23.0	

**Table 3:** Surgeon perceptions for each network.

Delay						
Network	*N*	25th percentile	50th percentile (Median)	75th percentile	Interquartile range	Kruskal–Wallis test (*P* value)
SINET (direct)	3	2.0	4.0	5.0	3.0	0.824
SINET (via FHU dedicated)	3	0.0	2.0	7.0	7.0	
JGN	3	2.0	3.0	4.0	2.0	
ARENA-PAC	3	2.0	3.0	3.0	1.0	
Stress						
SINET (direct)	3	3.0	6.0	7.0	4.0	0.162
SINET (via FHU dedicated)	3	4.0	5.0	8.0	4.0	
JGN	3	2.0	4.0	5.0	2.0	
ARENA-PAC	3	2.0	3.0	3.0	1.0	
Smoothness						
SINET (direct)	3	2.0	3.0	6.0	4.0	0.838
SINET (via FHU dedicated)	3	1.0	3.0	5.0	4.0	
JGN	3	3.0	4.0	5.0	2.0	
ARENA-PAC	3	3.0	3.0	4.0	1.0	

Notably, despite the SINET networks having lower latencies, surgeons’ perceptions of the delay and stress of operation were higher, and the smoothness of operation was comparable to JGN and ARENA-PAC networks. These findings, however, did not reach statistical significance.

### Telesurgical gastrectomy

**Figure 4:**
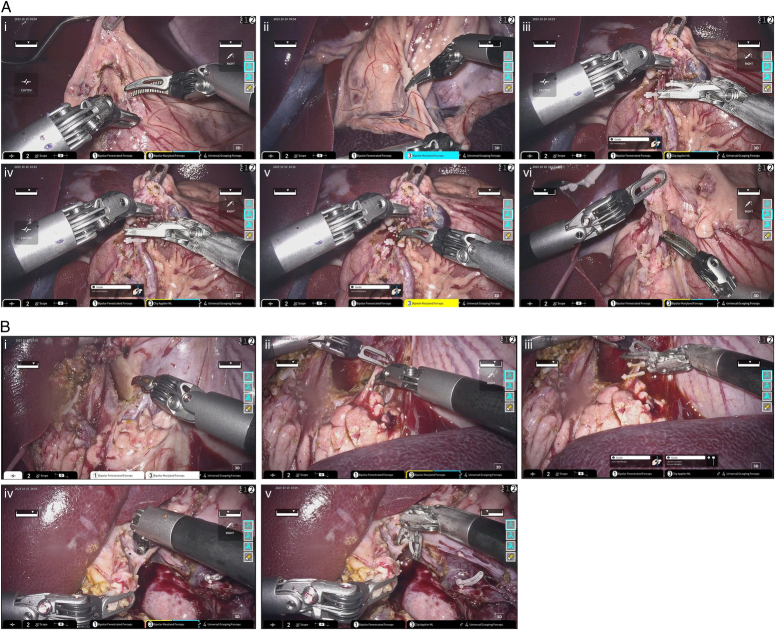
(A), Infra-pyloric dissection (station 6 lymph nodes) of the radical gastrectomy. Images (i)–(vi) involve isolation and ligation of the right gastroepiploic artery. (B) Suprapancreatic dissection of the radical gastrectomy. Images (i)–(iii) show the isolation and ligation of the coronary vein, and images (iv)–(v) detail the isolation and ligation of the right gastric artery originating from the left hepatic artery.

The components of each gastrectomy comprised mobilization of the stomach for resection, D2 lymphadenectomy, and reconstruction with Bilroth-I anastomosis. Minimal blood loss and no intraoperative complications were reported in all three procedures. The total operative time ranged from 151 to 203 min, with a median operative time of 176 min. Further breakdown of the surgical steps are as follows: median time (minutesand range) for lymphadenectomy was 58 min (57–65 min), gastric resection was 100 min (69–122 min), and anastomosis was 19 min (17–23 min). Images from the live porcine gastrectomies can be seen in Figure 4. The total latency time of all three telesurgical gastrectomies was 107 ms on the SINET (via FHU dedicated) network, as detailed above.

## Discussion

In 2001, Marescaux *et al*.^4^ reported the first transatlantic robot-assisted laparoscopic cholecystectomy performed over ~14 000 km on the ZEUS, Computer Motion, California Robotic System via connections using asynchronous transfer mode (ATM) technology with a mean delay of 155 ms. Since then, there have been remarkable technological advancements in telecommunications and robotic surgery. The recent Telesurgery Consensus Conference in Orlando, Florida, USA, in February 2024 provided deep insights into the current state-of-the-art robotic systems^20^. Recent trials in the last 2 years include telesurgical robotic prostatectomies on canine models and telecholecystectomies on artificial organ models across Japan, which have reported good outcomes^21,22^. The boundaries of telesurgery are continually challenged, with hospitals in China reporting ongoing telesurgical trials – including general surgery, urology, and gynecological cases – being performed on live human patients with the MicroPort MedBotTM Robotic Systems and KangDuo robotic system regionally^3,20,23,24^. This study represents the first report of a transnational telesurgical distal gastrectomy with D2 lymphadenectomy and intracorporeal Billroth-I anastomosis done in a live animal utilizing the new hinotori Surgical Robotic System. In our current study, we demonstrated that transnational radical D2 gastrectomy with telesurgery is feasible with no appreciable differences in surgeon performance and perception with network latencies of 107–132 ms.

This study revealed that it is feasible for a long-distance telesurgical gastrectomy to be performed safely. Robotic distal gastrectomy is a safe and acceptable oncologic procedure for gastric cancer, with established evidence supporting its safety and at least comparable postoperative outcomes compared to laparoscopic gastrectomy^25–30^. This telesurgical set-up to perform remote robotic distal gastrectomy had only been performed within Japan, ~30 km apart, demonstrating similar feasibility and safety, but has not been tested between countries^8^. The operations were performed with no intraoperative complications or significant technical issues. In addition to linking the surgeon’s cockpit and operation unit, the audio-visual link was established through third-party platforms for real-time communications. An annotation unit to facilitate interaction between the surgeon and operating staff was also available on the hinotori. This setup enables experts to provide real-time advice and support during the actual surgery. Surgeons were present at the operation unit as a fail-safe measure to take over the procedure locally if necessary. The experimental animals survived the procedures with no major complications and were well intraoperatively with stable hemodynamics without active intervention from the veterinary team prior to being euthanized. Recent studies by Inoue *et al*.^12^ and Kitadani *et al*.^31^ reporting median operative times for robotic gastrectomies in human subjects with the hinotori Surgical Robot System were 400 min and 282 min, respectively. Our previous study on a remote distal gastrectomy in two porcine models done regionally 30 km apart in Japan had total operation times from skin incision at 173 and 237 min, respectively (including console times of 160 and 225 min, respectively)^8^. The telesurgical robotic gastrectomies performed in our current trial ranged from 151 to 203 min, well within the acceptable operative timing. While there are still many barriers and ethical considerations in the adoption of long-range telesurgery into routine clinical practice, this study demonstrates the technical feasibility of performing complex oncologic robotic surgery across different countries in a live animal model.

A key consideration in utilizing telesurgery is the possible delay resulting from network latency. There is currently no consensus on the acceptable amount of latency time for remote telesurgery^32,33^. Latency studies with needle-driving exercises on robotic simulators by Xu *et al*.^34^ demonstrated the feasibility of remote telesurgery at a latency time of below 200 ms, while delaying instrument manipulation resulted in failure at task completion beyond 800 ms^35^. A review by Rayman *et al*.^36^ demonstrates that the effect of latency on telesurgical outcomes complies with Fitts’ law – with relatively small delays, the time of approach to the target is preserved, though as the delay increases, the approach becomes oscillatory and, in turn, increases completion time. This could account for the large variance in acceptable latency times across the literature. While feasibility has been demonstrated over a large range of latencies, there is a concern that as latencies increase, the difficulty and risks of complications increase and may not be completely compensated by the surgeon’s individual skill and experience. In our study, the latency times for all four wired networks ranged from 107 to 132 ms, well below the acceptable thresholds of latency. The standardized tasks were completed successfully across all international networks, with no significant difference in objective and subjective markers. Of note, surgeons’ perceptions of operative smoothness were found to be comparable in the SINET networks despite having marginally higher latency and delays which may be due to the order of networks tested, as the surgical tasks were carried out on the SINET network’s first, followed by the JGN and ARENA-PAC networks. The familiarity of the task and robotic platform may have improved with repetition and led to favorable perceptions of ease^37–40^. Furthermore, the difference between the fastest networks (SINET direct and via FHU dedicated) and the slowest network (ARENA-PAC) was 25 ms, which may have been too minute a difference to be perceived by the surgeons. According to our previous study^8^, the tolerable threshold of total latency in hinotori was determined as 100–125 ms. In addition, software updates by hinotori reduced the oscillation in the control of the robotic arms, possibly compensating for the differences in latency between networks. Subsequent gastrectomy procedures performed in three live porcine models were smoothly completed at a network latency of 107 ms.

With regard to clinical application, a recent review by Barba *et al*.^3^ on eight successful human and cadaveric telesurgical procedures have reported feasibility at latencies between 28 and 280 ms. Current technologies such as optic fibers have also been reviewed and shown to be able to send and receive 4K images under the 100 ms communication delay^41,42^, though this upper limit may be technically difficult to reduce as it is determined by the speed of light. Further technological advancements in the form of high-speed 5G wireless networks have also been explored, with Mohan *et al*.^43^ quoting a theoretical max speed of 5G up to 10G bps, bringing down current latencies from 0.27 s to 0.01 s. Pandav *et al*.’s^44^ review of successful telesurgical procedures done on 5G networks reported feasibility latencies between 28 and 264 ms^45^. However, the wireless network still needs to overcome a problem of fluctuation (variation in latency over time), and hence a combination of both wired and wireless 5G networks may be beneficial to enhance the safety of telesurgery^46^. Though in its nascent stages, there is anticipation with the further development of network options in improving the feasibility and safety of telesurgery.

Next, it is paramount to reconcile the technical aspect of maintaining cybersecurity during a telesurgical procedure. Healthcare institutions’ networks are susceptible to medical tampering, and it is a necessity to establish a network infrastructure with secure connections^47^. This study was conducted with the setup of private VPNs to minimize external interferences during the trials. Though encryption of data may increase network delay^2^, reducing the risk of any external interference that could disrupt ongoing telesurgery and pose threats to the patient’s safety remains a necessity.

The ethical and legal implications of telesurgery will also need to be considered^48,49^. A framework defining indications that would benefit patients in clinical practice, outweighing potential risks that are justifiable and mitigated, is crucial to guide the adoption of remote telesurgery. For instance, in advanced proximal gastric cancers invading the greater curve, spleen-preserving gastrectomy with lymphadenectomy of station 10 (splenic hilar) nodes is technically demanding and performed by few surgeons worldwide but has comparable oncologic outcomes and avoids complications and sequelae of a splenectomy^50^. In this setting, the station 10 lymph node dissection may be performed or proctored remotely by an expert, with a surgical team at the bedside available to salvage if major complications like massive bleeding are encountered. The party responsible for the consent of the patient and also legal obligations in the event of a negative outcome may vary between countries and needs to be determined on a case-specific basis. The benefits and clinical applicability of having remote guidance from an experienced procedurist have been recently demonstrated by Goel *et al*.^51^. A complex cardiovascular Laceration of the Anterior Mitral leaflet to Prevent Outflow Obstruction (LAMPOON) procedure – performed at only a few centers globally – was urgently required for a patient but was limited by travel restrictions for procedurists due to the COVID-19 pandemic. Remote telemonitoring and proctoring were done by a highly experienced procedurist, which aided in the successful completion of the procedure. The benefits of international cooperation in advancing procedural techniques and patient care should serve as encouragement in overcoming such ethical and legal considerations.

This study had several limitations. Firstly, the design of the study was aimed at testing the feasibility of transnational robotic telesurgery, and pre-existing high bandwidth, low-latency, and secure networks were selected. There was no systematic testing of increasing latency to establish an upper limit or threshold of latency that would affect surgeon performance or operative feasibility, given that it had already been demonstrated in a previous publication in established literature^8^. There could have been ethical challenges to evaluating these parameters in a live animal model. Next, testing was done only on the hinotori Surgical Robot System, a new robotic system, hence the results may not be generalizable to other surgical robots and will need to be tested on other robotic platforms. Lastly, this is a proof-of-principle preclinical study with porcine models. Though barriers exist, our encouraged results do pave the way for clinical application in the future.

## Conclusion

Transnational networks between Japan and Singapore were successfully established and tested using the hinotori Surgical Robot System. A network latency of 107–132 ms was identified but it did not affect surgeons’ perceptions or procedural timing in dry-box suturing tasks. We demonstrated the feasibility and success of the first transnational telesurgical robotic gastrectomy with D2 lymphadenectomy in a live animal model with no intraoperative complications.

## Ethical approval

All the procedures were performed under the approval of the Institutional Animal Care and Use Committee at Fujita Health University (APU22141-MD2). The handling of the porcine models was in accordance with the Regulations for the Management of Laboratory Animals at Fujita Health University.

## Consent

Not applicable.

## Source of funding

Not applicable.

## Author contribution

D.K.A.C.: conceptualization, methodology, validation, formal analysis, investigation, data curation, writing – original draft, writing – review and editing, visualization, supervision, and project administration; K.S., J.B.Y.S., and I.U.: conceptualization, methodology, validation, investigation, data curation, writing – review and editing, visualization, supervision, and project administration; W.-E.H.: validation, formal analysis, investigation, writing – original draft, writing – review and editing, visualization, and project administration; B.S.L.: software, resources, data curation, writing – review and editing, visualization, supervision, and project administration; K.S., A.S., and D.L.: resources, data curation, writing – review and editing, supervision, and project administration; S.S. and T.H.: resources, data curation, writing – review and editing, supervision, and project administration; J.M., M.O., H.A., T.T., H.S., and M.O.: software, resources, data curation, writing –review and editing, supervision, and project administration; M.Y. and H.K.: software, resources, data curation, writing – review and editing, visualization, supervision, and project administration; S.P.G. and Y.X.: software, resources, data curation, writing – review and editing, and project administration; G.W.K.: investigation, data curation, writing – review and editing, supervision, and project administration.

## Conflicts of interest disclosure

M.N., S.S., K.S., I.U., and K.S. have no commercial associations or financial involvement that might be construed as a conflict of interest in connection with the submitted article. I.U. has received lecture fees from Intuitive Surgical, Inc., outside of the submitted work. I.U. has been funded by Medicaroid Corporation in relation to the Collaborative Laboratory for Research and Development in Advanced Surgical Technology, Fujita Health University. K.S. has been funded by Sysmex Corporation in relation to the Collaborative Laboratory for Research and Development in Advanced Surgical Intelligence, Fujita Health University. K.S. has also been funded by Medtronic, Inc., outside of the submitted work.

## Research registration unique identifying number (UIN)

1. Name of the registry: not applicable.
2. Unique identifying number or registration ID: not applicable.
3. Hyperlink to your specific registration (must be publicly accessible and will be checked): not applicable.

## Guarantor

Daryl Kai Ann Chia, Koichi Suda, Jimmy Bok Yan So, and Ichiro Uyama.

## Data availability statement

All data underlying the results are available as part of the article and no additional source data are required.

## Provenance and peer review

This paper was not invited.

## Supplementary Material

SUPPLEMENTARY MATERIAL
