# Supplementary figures and images for: Feasibility of long-range telesurgical robotic radical gastrectomy in a live porcine model
Source: Int J Surg. 2024 Nov 22;110(12):7720–8. doi: 10.1097/JS9.0000000000002151 (PMC11634161; doi:10.1097/JS9.0000000000002151)

**APPENDIX**


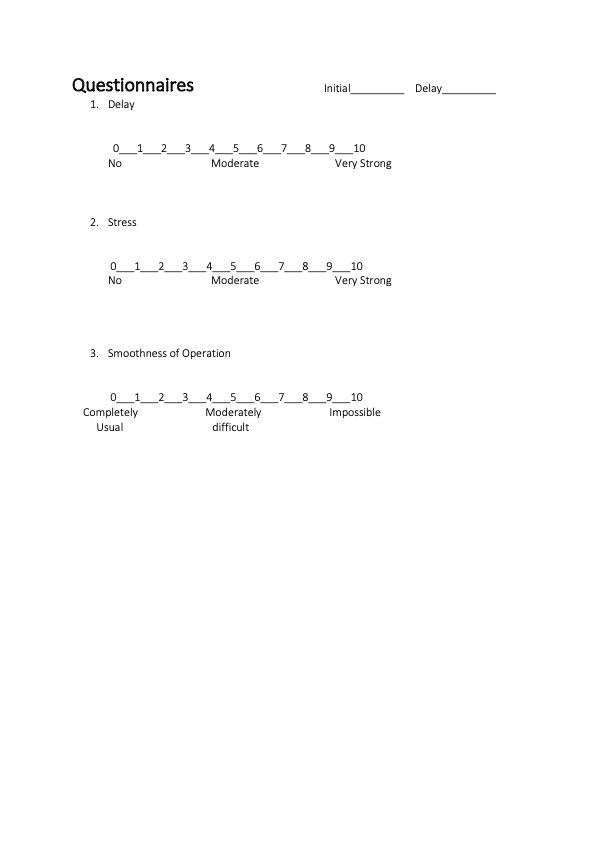


Appendix Figure 1. Sample surgeon questionnaire for standardized suture dry task

Supplement: SUPPLEMENTARY MATERIAL [file js9-110-7720-s001.docx]
