# Supplementary material for: Feasibility of long-range telesurgical robotic radical gastrectomy in a live porcine model
Source: Int J Surg. 2024 Nov 22;110(12):7720–8. doi: 10.1097/JS9.0000000000002151 (PMC11634161; doi:10.1097/JS9.0000000000002151)
Supplement: SUPPLEMENTARY MATERIAL [file js9-110-7720-s002.doc]

**The ARRIVE Guidelines Checklist**

**Animal Research: Reporting *In Vivo* Experiments**

Carol Kilkenny^1^, William J Browne^2^, Innes C Cuthill^3^, Michael Emerson^4^ and Douglas G Altman^5^

*^1^The National Centre for the Replacement, Refinement and Reduction of Animals in Research, London, UK, ^2^School of Veterinary Science, University of Bristol, Bristol, UK, ^3^School of Biological Sciences, University of Bristol, Bristol, UK, ^4^National Heart and Lung Institute, Imperial College London, UK, ^5^*Centre for Statistics in Medicine, University of Oxford, Oxford, UK

|  | **Item** | **Recommendation** | **Section/ Paragraph** |
| --- | --- | --- | --- |
| **TITLE** | 1 | Provide as accurate and concise a description of the content of the article as possible | Title |
| **ABSTRACT** | 2 | Provide an accurate summary of the background, key methods, including the details of the species of animals used, principle findings and conclusions of the study | Abstract |
| **INTRODUCTION** |  |  |  |
| - **Background** | 3 | a. Include sufficient scientific background (including relevant references to previous work) to understand the motivation and context for the study, and explain the experimental approach and rationale  b. Explain how and why the animal species and model being used can address the scientific objectives and, where appropriate, the study’s relevance to human biology | Paragraph 1  Paragraph 2 |
| - **Objectives** | 4 | Clearly describe the primary and any secondary objectives of the study, or specific hypotheses being tested | Paragraph 3 |
| **METHODS** |  |  |  |
| - **Ethical statement** | 5 | Indicate the nature of the ethical review permissions, relevant licenses (e.g. Animal [Scientific Procedures] Act 1986), and national or institutional guidelines for the care and use of animals, that cover the research | Paragraph 9 |
| - **Study design** | 6 | For each experiment, give brief details of the study design including:  a. The number of experimental and control groups  b. Any steps taken to minimize the effects of subjective bias when allocating animals to treatment (e.g. randomization procedure) and when assessing results (e.g. if done, describe who was blinded and when).  c. The experimental unit (e.g. a single animal, group or cage of animals).  A time-line diagram or flow chart can be useful to illustrate how complex study designs were carried out | Paragraphs 7-9  Not applicable  Paragraphs 7-9  Not applicable |
| - **Experimental procedures** | 7 | For each experiment and each experimental group, including controls, provide precise details of all procedures carried out. | Paragraphs 7-9 |
| - **Experimental animals** | 8 | a. Provide details of the animals used, including species, strain, sex, developmental stage (e.g. mean or median age plus age range) and weight (e.g. mean or median weight plus weight range)  b. Provide further relevant information such as the source of animals, international strain nomenclature, genetic modification status (e.g. knock0out or transgenic), genotype, health/ immune status, drug or test naïve, previous procedures, etc. | Paragraphs 7-9 |
| - **Housing and husbandry** | 9 | Provide details of:  a. Housing  b. Husbandry conditions  c. Welfare-related assessments and interventions that were carried out prior to, during, or after the experiment | Paragraphs 7-9 |
| - **Sample size** | 10 | a. Specify the total number of animals used in each experiment, and the number of animals in each experimental group.  b. Explain how the number of animals were arrived at. Provide details of any sample size calculation used.  c. Indicate the number of independent replications of each experiment, if relevant | Paragraphs 7-9 |
| - **Allocating animals to experimental groups** | 11 | a. Give full details of how animals were allocated to experimental groups, including randomization or matching if done  b. Describe the order in which the animals in the different experimental groups were treated and assessed. | Not applicable |
| - **Experimental outcomes** | 12 | Clearly define the primary and secondary experimental outcomes assessed. | Paragraphs 7-9 |
| - **Statistical methods** | 13 | a. Provide details of the statistical methods used for each analysis.  b. Specify the unit of analysis for each dataset (e.g. single animal, group of animals, single neuron).  c. Describe any methods used to assess whether the data met the assumptions of the statistical approach | Paragraph 10 |
| **RESULTS** |  |  |  |
| - **Baseline data** | 14 | For each experimental group, report relevant characteristics and health status of animals prior to treatment or testing. | Methods Paragraphs 8-9 |
| - **Numbers analyzed** | 15 | a. Report the number of animals in each group included in each analysis.  b. If any animals or data were not included in analysis, explain why | Methods Paragraphs 8-9 |
| - **Outcomes and estimation** | 16 | Report the results for each analysis carried out, with a measure of precision | Paragraph 6  Figures 5(a) & (b) |
| - **Adverse events** | 17 | a. Give details of all important adverse events in each experimental group.  b. Describe modifications to the experimental protocols made to reduce adverse events | Paragraph 6 |
| **DISCUSSION** |  |  |  |
| - **Interpretation/scientific implications** | 18 | a. Interpret the results, taking into account the study objectives and hypothesis, current theory and other relevant studies in literature  b. Comment on the study limitations including any potential sources of bias, any limitations of the animal model, and the imprecision associated with the results  c. Describe any implications of your experimental methods or findings for the replacement, refinement and reduction (the 3Rs) of the use of animals in research | Throughout  Paragraph 6  Paragraph 6 |
| - **Generalisability/translation** | 19 | Comment on whether, and how, the findings of this study are likely to translate to other species or systems, including any relevance to human biology. | Throughout |
| - **Funding** | 20 | List all funding sources (including grant number) and the role of the funder(s) in the study. | Not applicable |

References:

1. Kilkenny C, Browne WJ, Cuthill IC, Emerson M, Altman DG (2010) Improving Bioscience Research Reporting: The ARRIVE Guidelines for Reporting Animal Research. *PLoS Biol* 8(6): e1000412. doi:10.1371/journal.pbio.1000412
2. Schulz KF, Altman DG, Moher D, the CONSORT Group (2010) CONSORT 2010 Statement: updated guidelines for reporting parallel group randomized trials. *BMJ* 340:c332.
